# A Novel Flexible Full-Cell Lithium Ion Battery Based on Electrospun Carbon Nanofibers Through a Simple Plastic Package

**DOI:** 10.1186/s11671-018-2788-7

**Published:** 2018-11-20

**Authors:** Xueyang Shen, Ziping Cao, Miao Chen, Jinya Zhang, Dong Chen

**Affiliations:** 1 College of Electronic and Optical Engineering and College of Microelectronics, Nanjing University of Posts and Telecommunications, Nanjing, 210046 China; 2 College of Telecommunication and Information Engineering, Nanjing University of Posts and Telecommunications, Nanjing, 210046 China; 3 College of Mechanical and Electrical Engineering, Jinling Institute of Technology, Nanjing, 21169 China; 4 Narada Power Source Co., LTD, Hangzhou, 310000 Zhejiang China

**Keywords:** Lithium batteries, Flexible, Anode, Carbon nanofiber, Full cell

## Abstract

The paper reports a novel flexible full-cell lithium ion battery (LIB) through a simple plastic package method. Carbon nanofibers (CNFs) are synthesized by electrospinning technology and the subsequent carbonation process. The CNFs with three-dimensional interconnected fibrous nanostructure exhibit a stable reversible capacity of 412 mAh g^−1^ after 100 cycles in the half-cell testing. A full cell is assembled by using CNF anode and commercial LiCoO_2_ cathode, and it displays good flexibility and lighting LED ability. The aggregate thickness of the constructed full-cell LIB is approximately 500 μm, consisting of a CNFs/Cu film, a separator, a LiCoO_2_/Al film, electrolyte, and two polyvinyl chloride (PVC) films. The structure, morphology, and the electrochemical performances of electrospun CNFs and LiCoO_2_ electrodes are analyzed in details.

## Background

In recent years, flexible energy storage devices have drawn special attention due to their portability, foldability, small space occupation, and shape diversification [1–4]. In particular, there has been an urgent requirement for advanced bendable lithium ion batteries (LIBs) along with the rapid development of flexible electronics. Comparing with other energy systems, the LIBs have several advantages like high energy density and cyclic stability, low self-discharge, non-memory effect, and environmental friendliness [5–7].

Up to now, some progress has been made in flexible LIBs mainly in terms of flexible electrodes. Xue and coworkers reported self-standing porous LiCoO_2_ nanosheet arrays as 3D cathodes for flexible LIBs, which exhibited a high reversible capacity of 104.6 mAh g^−1^at 10 C rate after 1000 cycles [8]. Deng et al. fabricated a flexible electrode by assembling the 3D ordered macroporous MoS_2_@C nanostructure on carbon cloth [9]. It was proved that such unique nanostructures made great contribution to the superior cycling stability when used as anode for LIBs. In addition to the present studies of flexible electrodes (cathodes and anodes), a novel kind of highly flexible separator based on hydroxyapatite nanowires is reported, making it promising for the application in flexible LIBs [10].

In general, coin cells were assembled in order to evaluate the charge–discharge performance of the above electrodes [11–14], while in this case, electrochemical testing of such electrodes under bending condition is hard to achieve through half-cell fabricating. Therefore, some studies have been involved in full cells in order to evaluate the performance of the flexible LIBs. A research group from Stanford University reported a new structure of thin, flexible LIBs [15]. In this work, the current collectors and LIB materials were integrated onto a single sheet of paper, exhibiting robust mechanical flexibility and high energy density. Another flexible LIB based on all-solid-state materials through a polydimethylsiloxane (PDMS) packaging process was explored by Koo et al. [16]. The bendable LIB was integrated with a light-emitting diode (LED) to form an all-in-one flexible electronic system. Despite the excellent performance of the abovementioned flexible LIBs, the complex preparation processes are major drawbacks for their practical use in commercial LIBs.

Carbon nanofibers (CNFs) have drawn attention for their unique advantages in energy devices. When used as anodes for LIBs, CNFs with three-dimensional interconnected fibrous nanostructure may shorten the diffusion path for lithium ions as well as provide good stability [17, 18]. In recent years, CNFs have been primarily exploited as supporting frameworks to load active materials (SnO_2_, Si, MnO_x_, etc.) [19–21]. Electrospinning and followed thermal treatment is a simple and low-cost approach to prepare CNFs. The diameter and morphology of CNFs can be flexibly controlled by the spinning conditions.

**Fig. 1:**
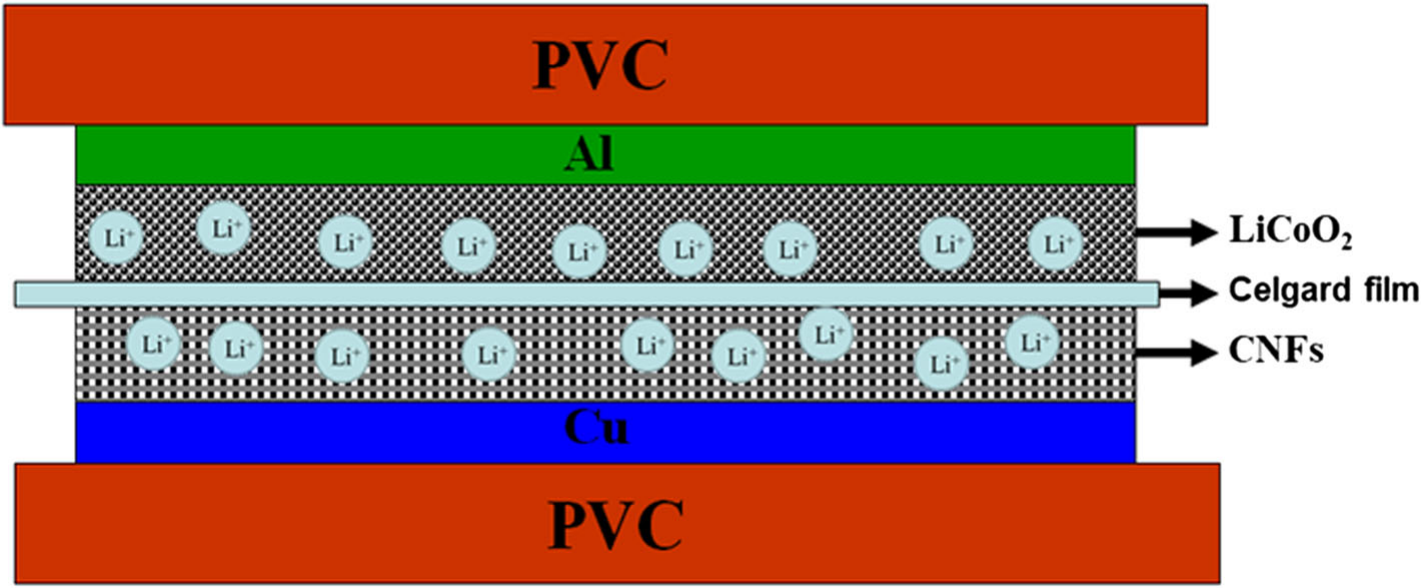
Schematic illustration for the inner structure of the flexible thin-film LIB

Herein, we construct a stacked structure of flexible thin-film LIB through a simple plastic package method. Figure 1 shows the schematic of the fabricated flexible full cell, consisting of the carbon nanofibers (CNFs)/Cu film (anode), a separator, the LiCoO_2_/Al film (cathode), electrolyte, and polyvinyl chloride (PVC) films. CNFs were prepared through an electrospinning method and the subsequent carbonation process. PVC film serves as a flexible substrate and encapsulation material in consideration of its light weight and good flexibility. LiCoO_2_/Al film and CNFs/Cu film can be obtained through a coating method, which are used as the positive and negative electrode, respectively. A laminator is introduced to complete the encapsulation of the flexible LIBs. Apart from the reported packaging method, the laminator is easy to operate, low in energy consumption. It is especially suitable for the packaging of such multi-layer film stacked flexible LIBs. The study aims at assembling a novel structure of a flexible LIB by means of full cell and investigating its charge–discharge performance under bending.

## Methods

### Synthesis of CNFs

An electrospinning method was used to synthesize the CNFs. Two grams of polyacrylonitrile (PAN, Mw = 150,000, J&K Scientific LTD. N) was added in 20 mL of *N*, *N*-dimethylformamide (DMF, Beijing Chemical Works) under magnetic stirring at 50 °C until complete dissolution. The electrospinning process was provided by a variable high-voltage power supply (SS-2534, Beijing Ucalery Company). The applied working voltage, flow rate, and needle-to-collector distance were 20 kV, 0.6 mL h^−1^, and 15 cm, respectively. The electrospun PAN fibers were collected using Al foil and heated to 280 °C for 1 h in an air environment with a heating rate of 5 °C min^− 1^. Finally, they were carbonized at 700 °C for 2 h in argon atmosphere (heating rate was 2 °C min^−1^).

### Fabrication of LiCoO_2_/CNF Flexible Full Cell

Here are three steps for assembling a novel structure of flexible LIB through the plastic package method.

Firstly, the preparation of two flexible electrodes: the positive electrode was prepared by pasting slurries onto an aluminum current collector with a doctor blade method. The slurry was made by mixing active materials LiCoO_2_, carbon black (Super P), and polyvinylidene fluoride (PVDF) in a weight ratio of 90:5:5. The negative electrode was processed in the same technique apart from the following three points: the copper foil was applied as the current collector, the active anode material was as-prepared CNFs instead, and the weight ratio of CNFs, Super P, and PVDF is 80:10:10. Subsequently, the electrode sheets were firstly dried at room temperature and then transferred to an oven set at 80 °C for 12 h. Positive and negative electrodes were then cut into rectangles (5 mm long, 5 mm wide) and dried for additional 12 h under vacuum at 120 °C.

Secondly, the plastic package process using a laminator: the construction of the bendable LIB started with an appropriate cutting size of a PVC film layer, a CNFs/Cu anode, a separator, a LiCoO_2_/Al cathode, and another PVC film layer were stacked in order. Then, three sides of the above multilayer-structure cell were encapsulated by a laminator.

Thirdly, the injection of electrolyte was conducted in an Ar-filled glove box (the concentrations of moisture and oxygen below 1 ppm). The last unclosed side of the assembled full cell was encapsulated with seal gum. The electrolyte was 1 mol L^−1^ LiPF_6_/DMC + DEC + EC solution (1:1:1 in volume); the separator was Celgard 2300 film.

### Characterization

X-ray diffraction (XRD) patterns were measured by an Ultima IV diffractometer with Cu Kα radiation at a scan rate of 8° min^−1^ from 10° to 80°. Scanning electron microscope (SEM) images were observed with HITACHI SU-8010 and FEI QUANTA 6000 electron microscope.

### Electrochemical Test

The electrochemical performance of LiCoO_2_ cathode and CNF anode was tested using coin-type cells (CR2025) assembled in an argon-filled glove box. Galvanostatic charge/discharge tests were carried out by a LAND2001 CT battery tester. Electrochemical impedance spectroscopy (EIS) measurements were performed on an electrochemical workstation (CHI 660 D, CHI Company) under a frequency range between 100 kHz and 0.1 Hz with an applied voltage of 10 mV.

## Results and Discussion

**Fig. 2:**
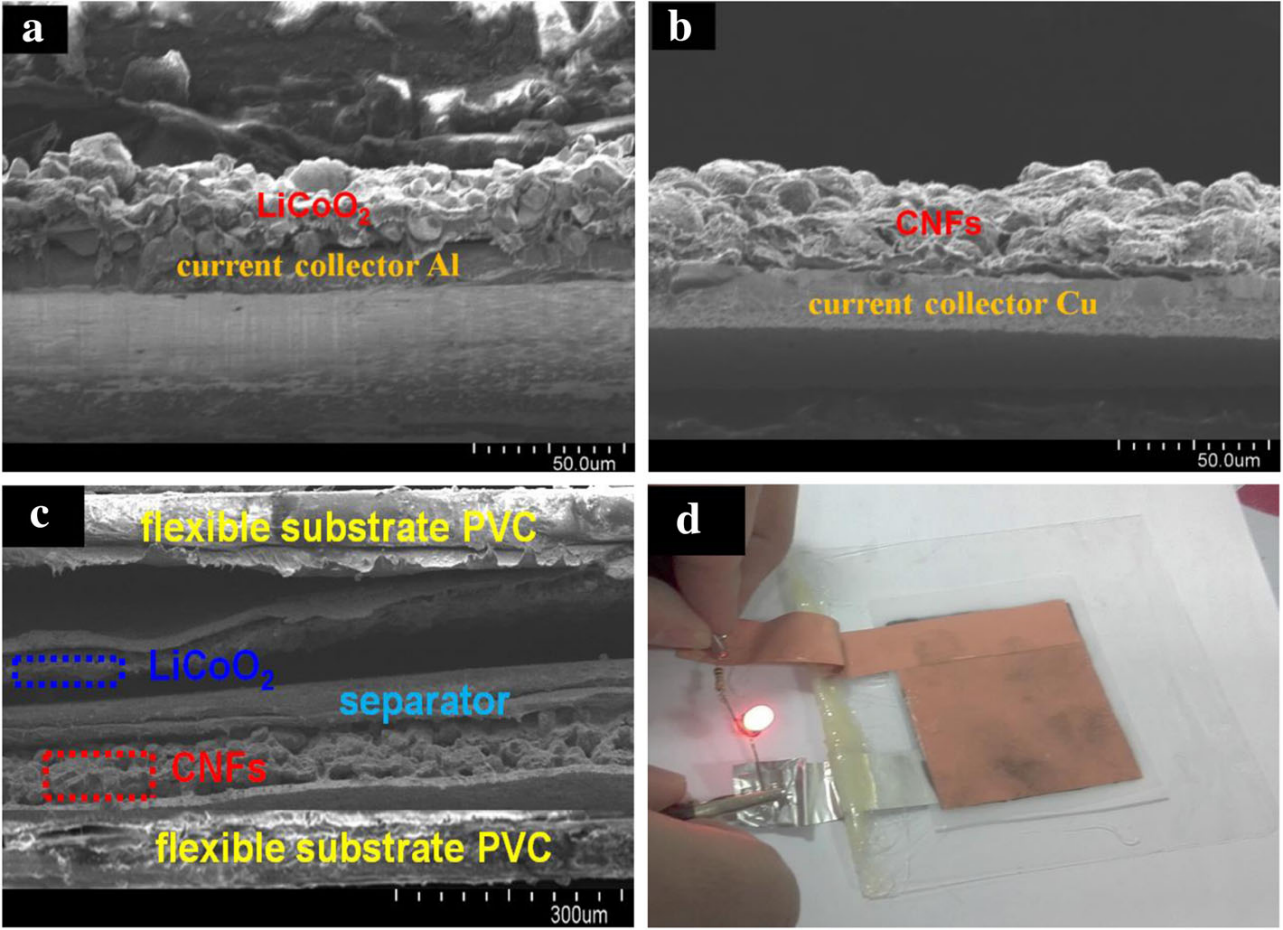
The cross-sectional images of **a** LiCoO_2_/Al film (cathode), **b** CNFs/Cu film (anode) and **c** flexible full cell, **d** photograph of LED lightened by the assembled full LIB

The cross-sectional images of LiCoO_2_/Al film (cathode), CNFs/Cu film (anode), and flexible full cell are shown in Fig. 2. Figure 2a indicates the tight combination between LiCoO_2_ and current collector via the doctor-blade coating process. Figure 2b reveals a successful coating of approximately 25-μm-thickness CNFs on the surface of the Cu current collector. We assembled a full LIB device based on the as-prepared LiCoO_2_ cathode and CNF anode. Figure 2c shows the cross section of the sandwich-shaped architectures encapsulated within two pieces of PVC films. A PVC film substrate, an anode current collector (Cu), a carbon anode (CNFs), a separator (Celgard 2300 film), a cathode (LiCoO_2_), a cathode current (Al), and a PVC film substrate are sequentially stacked in multiple layers. The aggregate thickness of the full cell is approximately 500 μm. In Fig. 2d, the assembled full cell can light up the LED continually when served as a power supply, showing promising prospect for application in future flexible electronic devices.

**Fig. 3:**
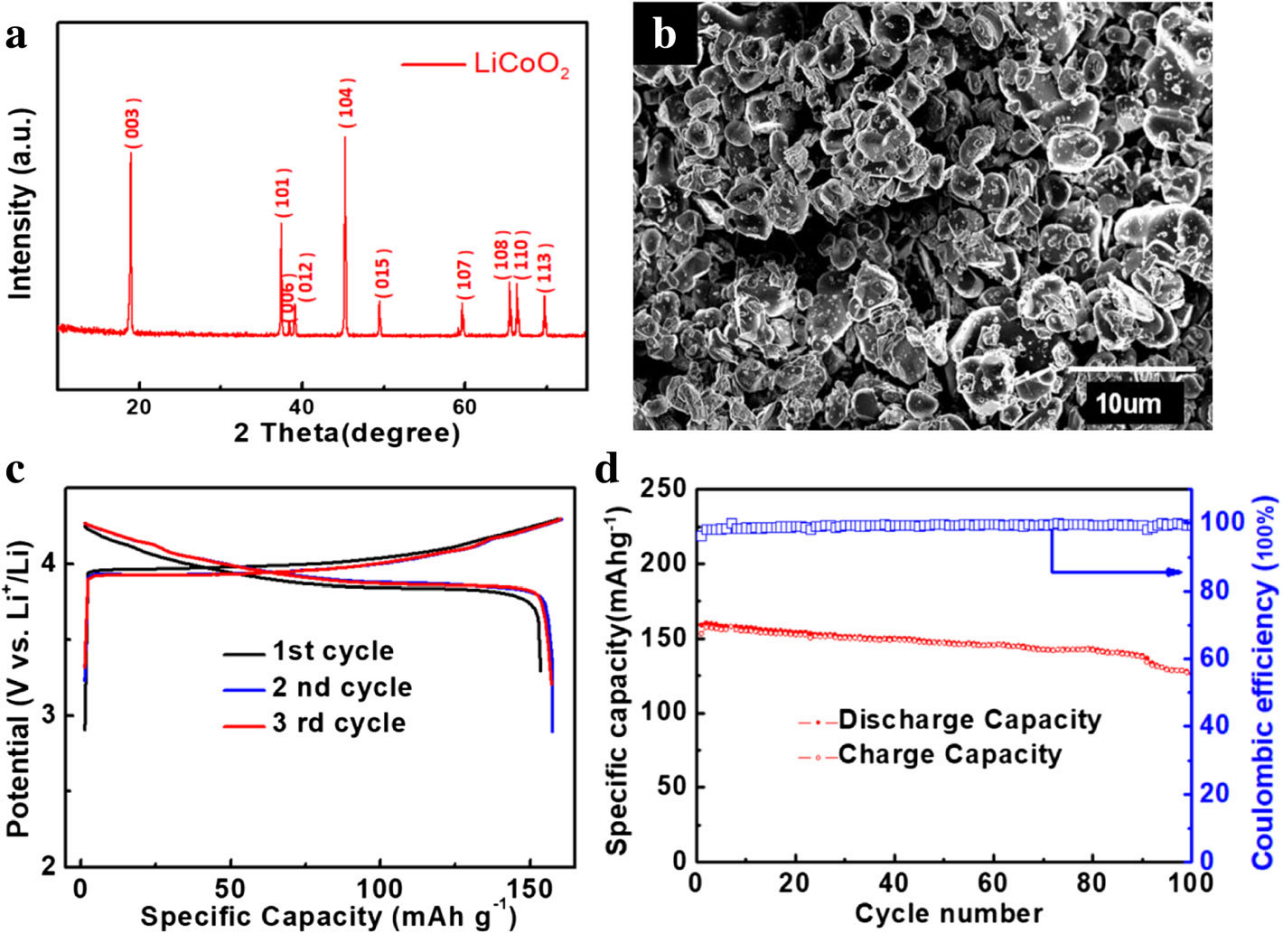
**a** XRD pattern, **b** SEM image, **c** charge–discharge curves, and **d** cycling performance of LiCoO_2_ cathode

As shown in Fig. 3a, the XRD pattern reveals that the crystal structure of LiCoO_2_ is in good agreement with a layered structure (JCPDS No. 44–145) [22]. The peaks appearing at 2 *θ* = 18.9°, 37.4°, 38.4°, 39°, 45.2°, 49.5°, 59.6°, 65.4°, 66.3°, and 69.7° can be indexed to the hexagonal LiCoO_2_ with the planes of (003), (101), (006), (012), (104), (105), (107), (108), (110), and (113), respectively [23]. The SEM observation (Fig. 3b) of LiCoO_2_ displays a laminate type of structure with well distribution, together with an average particle size of 5 μm. The as-prepared LiCoO_2_/Al film is tested as a cathode against lithium for half-cell performance measurement at a voltage window ranging from 3.2 to 4.3 V. Figure 3c shows the galvanostatic charge–discharge curves of the LiCoO_2_ electrode measured at 0.5 C rate. In the first cycle, discharge/charge capacities of 153.5 mAh g^−1^ and 159.2 mAh g^−1^ are obtained, corresponding to a coulombic efficiency of 96.4%. The long potential plateau near 4 V can be attributed to the reversible two-phase reaction, which is a typical property of the layered LiCoO_2_ phase [24, 25]. In the subsequent cycles, the positions of the curves have no apparent shift, implying good reversibility. The cycling performance of LiCoO_2_ cathode is shown in Fig. 3d, which exhibits a reversible capacity of 126.3 mAh g^−1^ after 100 cycles.

**Fig. 4:**
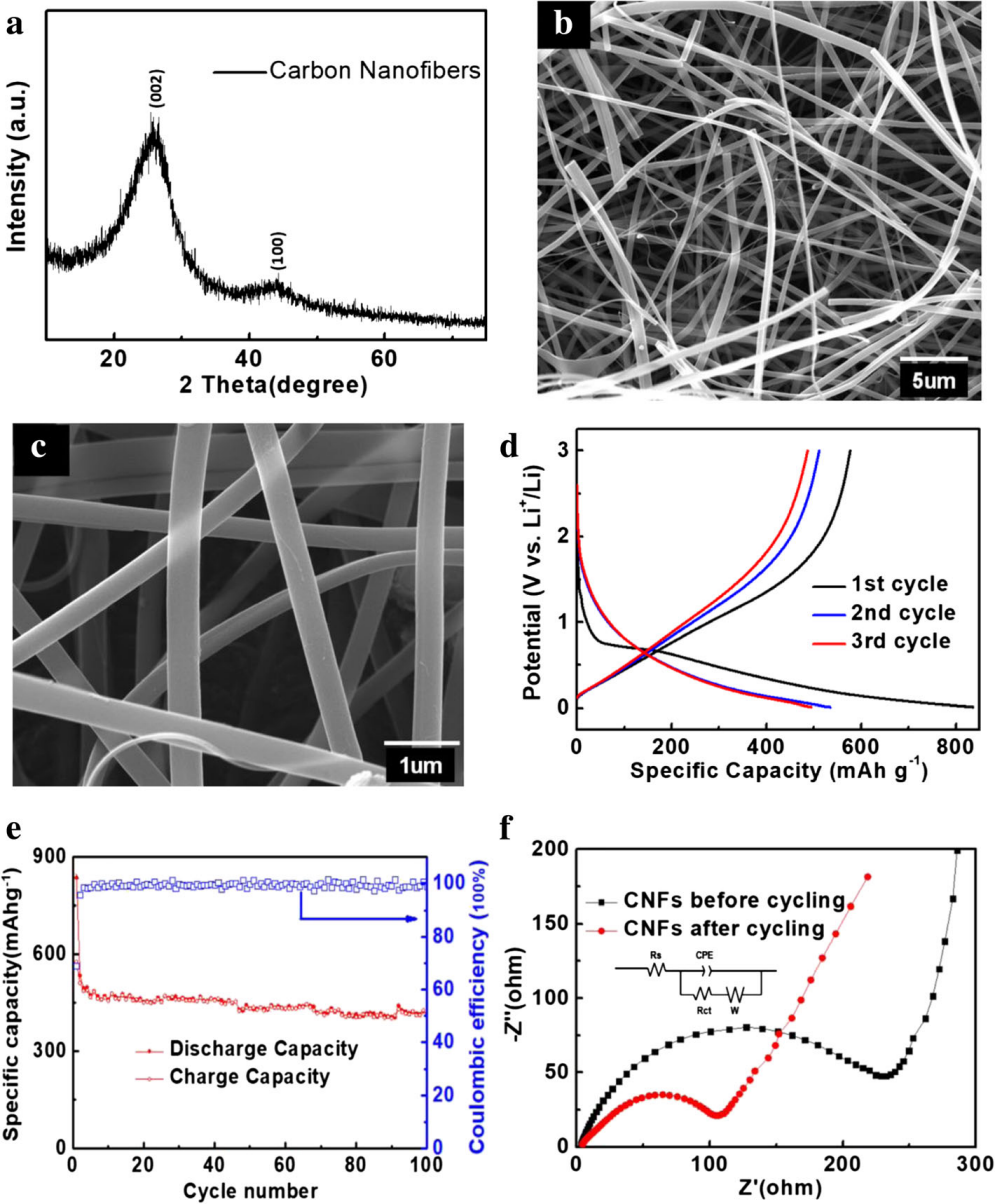
**a** XRD pattern, **b**, **c** SEM images, **d** charge–discharge curves, and **e** cycling performance of CNFs anode and **f** Nyquist plots at the OCP and the equivalent circuit for electrospun CNF electrodes before and after discharge/charge cycles

XRD pattern of the electrospun CNFs is shown in Fig. 4a. Two peaks located at 2θ = 23° and 42° can be indexed to the (002) and (100) planes of carbon, respectively [26, 27]. The weak and broad peaks indicate the low crystallinity of the obtained CNFs, which is corresponding to the amorphous carbon structure [28]. To get more insight into the morphology of CNFs, SEM observation is given in Fig. 4b, c. It is clear that the CNFs display a three-dimensional (3D) interconnected fibrous nanostructure through the electrospinning process. The carbon nanofibers are randomly well-distributed, and the diameters range from 300 to 400 nm.

In order to investigate the electrochemical performance of CNF anode, galvanostatic charge–discharge tests were carried out between 0.01 and 3 V at a current density of 100 mA g^−1^ as shown in Fig. 4d. The CNF anode exhibits initial discharge/charge capacities of 836 and 576.7 mAh g^−1^, respectively. The value is higher than the theoretical capacity (372 mAh g^−1^) of graphitic carbon. This phenomenon is common in the non-graphitic carbonaceous materials synthesized at low temperatures (500–1000 °C) [29]. This can be described to the formation of Li_*x*_C_6_ (where *x* is about 1.2–3.0) during the intercalation process, rather than LiC_6_ in graphitic carbon [30, 31]. There is a plateau near 0.7 V in the first discharge curves, but it disappears in the subsequent cycles. It is the main reason for the initial irreversible capacity of 259.3 mAh g^−1^, which is caused by the solid electrolyte interface (SEI) formation and corrosion-like reaction of Li_*x*_C_6_ [32]. From the second cycling, it is clearly seen that the major contribution to the reversible capacity occurs below 0.4 V.

The cycling performance of CNF anode at a current density of 100 mA g^−1^ is shown in Fig. 4e. The CNFs hold a reversible capacity of 412 mAh g^−1^ after 100 cycles, which is higher than the commercial MCMB anode materials under the same experimental condition. A high coulombic efficiency of nearly 100% is achieved except for the first cycle. The main reason for the modified cyclic stability and reversible capacity is the interconnected 3D networks of the electrospun carbon nanofibers. Such framework provides enough space for the lithium intercalation/deintercalation reactions, as well as facilitates the diffusion of lithium ions and electrolyte. Moreover, the fibrous carbon with good structural stability and electrical conductivity is also beneficial to the improved cyclic reversibility.

Electrochemical impedance spectra (EIS) measurement was carried out before and after charge/discharge cycles so as to demonstrate the kinetic features of CNF anode. In Fig. 4f, the Nyquist plots of both anodes contain one semicircle in the high-frequency region and an inclined line in the low-frequency region [33, 34]. The intercept on the *Z*_real_ axis can be assigned to the electrolyte resistance (*R*_s_), while the semicircle is ascribed to electron transfer resistance (*R*_ct_). The slope line is corresponding to Warburg (*R*_w_) over Li^+^ diffusion in the solid materials [35, 36]. The *R*_ct_ of CNF anode is 237.4 Ω for the fresh cell. After cycling for 100 cycles, the value of *R*_ct_ decreases to 108.2 Ω, indicating a higher electrochemical reactivity. The improvement in kinetics of the CNF anode can be attributed to activation of the anode after the charge/discharge processes.

## Conclusions

A novel flexible full-cell LIB is constructed through a simple plastic package method, consisting of a CNFs/Cu film, a separator, a commercial LiCoO_2_/Al film, electrolyte, and two polyvinyl chloride (PVC) films. Carbon nanofibers (CNFs) are synthesized by electrospinning and the subsequent carbonation process. The CNFs with three-dimensional interconnected fibrous nanostructure exhibit a stable reversible capacity of 412 mAh g^−1^ after 100 cycles in the half-cell testing. The cycling performance of commercial LiCoO_2_ cathode shows a reversible capacity of 126.3 mAh g^−1^. PVC film serves as a flexible substrate and encapsulation material. The full-cell LIB can light up the LED continually when served as a power supply, indicating good flexibility and power supply ability.

## Abbreviations

CNFs: Carbon nanofibers
DMF: *N*, *N*-Dimethylformamide
EIS: Electrochemical impedance spectroscopy
LED: Light-emitting diode
LIB: Lithium ion battery
PAN: Polyacrylonitrile
PDMS: Polydimethylsiloxane
PVC: Polyvinyl chloride
PVDF: Polyvinylidene fluoride
*R*_ct_: Electron transfer resistance
*R*_s_: Electrolyte resistance
SEI: Solid electrolyte interface
SEM: Scanning electron microscope
XRD: X-ray diffraction
